# Cardiac Autonomic Functions in Obese Children

**DOI:** 10.4274/jcrpe.v3i2.131

**Published:** 2011-06-08

**Authors:** Mehmet Emre Taşçılar, Mehmet Yokuşoğlu, Mehmet Boyraz, Oben Baysan, Cem Köz, Ruşen Dündaröz

**Affiliations:** 1 Departments of Pediatrics Division of Endocrinology, Gulhane Military Medical Academy, Ankara, Turkey; 2 Departments of Pediatrics Division of Cardiology, Gülhane Military Medical Academy, Ankara, Turkey; 3 Department of Pediatrics, Bezmialem Vakıf University, İstanbul, Turkey

**Keywords:** Childhood obesity, cardiac autonomic function, heart rate variability, power spectral analysis, time-domain analysis

## Abstract

**Objective:** The autonomic nervous system is assumed to have a role in the pathophysiology of obesity. In this study, we evaluated the autonomic system by measuring heart rate variability (HRV) in obese children.

**Methods: **Thirty-two obese and 30 healthy children (mean ages: 11.6±2.0 years and 11.0±2.9 years, respectively) were enrolled in the study. Obesity was defined as a body mass index higher than 97th percentile for age- and gender-specific reference values. All participants were free of any disease and none of them was receiving any medication. Twenty-four-hour ambulatory electrocardiographic recordings were obtained and the time-domain and frequency-domain indices of HRV were analyzed. The study group was evaluated with respect to insulin resistance by HOMA-IR values.

**Results:** A significant decrease in calculated HRV variables was observed in obese children as compared to controls. The HRV alteration was found in both time-domain and frequency-domain parameters. The subgroup analysis of the study group revealed a significant decrease in all investigated HRV parameters in the insulin-resistant obese children compared to the non-insulin-resistant obese ones.

**Conclusions:** Our results indicate that HRV is decreased in obese children, which implies parasympathetic withdrawal and sympathetic predominance. A marked decrease in HRV was observed in insulin-resistant obese children compared to their non-insulin-resistant counterparts. We propose that autonomic imbalance pertaining especially to insulin resistance may be involved in the pathogenesis of obesity in pediatric patients

**Conflict of interest:**None declared.

## INTRODUCTION

In today’s world, obesity is a prevalent disease in both developed and developing countries (1). It is a well-documented fact that obese people suffer from increased mortality risk due to cardiovascular complications (2,3,4). The assumption that the sympathoadrenal system plays a major role in the pathophysiology of obesity through regulation of energy expenditure is widely accepted (5). Chronic sympathetic overstimulation and increased catecholamine levels have been incriminated in obesity (6,7). Some authors have suggested that obese individuals demonstrate altered reactions to stressors (8). Equally unresolved is the question of whether the aberrations in the sympathetic system contribute to obesity or they are rather a consequence of it (9). 

While obesity in childhood is also known to be highly associated with multiple co-morbidities such as hypertension, dyslipidemia, reduced insulin sensitivity and alterations in large and small blood vessels (10), the number of studies that analyze the autonomic functions in obese pediatric populations is still limited.

Heart rate variability (HRV), defined as degree of fluctuation of the beat-to-beat differences in cardiac rhythm, is known to be a reliable, noninvasive marker of autonomic nervous system activity (11,12). The loss of this beat-to-beat variability is indicative of various diseases (13,14,15,16,17,18). Detection of such changes, especially for the evaluation of autonomic nervous system functions, may be used as a marker of underlying pathology. In this case-control study, we aimed to investigate the cardiac autonomic function by using time-domain and frequency-domain parameters of HRV in children with obesity

## MATERIALS AND METHODS

The study group consisted of 32 consecutive obese children (20 male, 12 female) with a mean age of 11.6±2.0 years and the control group included 30 age- and gender-matched healthy children (19 male, 11 female; mean age: 11.0±2.9 years). Control subjects were chosen from among those who presented to our outpatient clinic for routine check-up. To exclude the presence of any concomitant disease, a detailed medical history was obtained and a physical examination (including evaluation for syndromes and endocrine diseases) was performed. Complete blood count and blood chemistry, consisting of fasting insulin, thyroid function tests and serum cortisol, were evaluated to rule out any underlying pathology.  Children with obesity-related entities such as syndromic (Prader-Willi syndrome, Laurence-Moon-Biedl syndrome, etc.) and endocrine (Cushing’s syndrome, hypothyroidism, etc.)  diseases were excluded from the study. The study protocol was reviewed and approved by the local ethics committee.

Weight and height measurements were done with the children wearing light clothes and no shoes. Standing height (cm) was measured to the nearest 0.1 cm with a Harpenden fixed stadiometer and body weight (kg) on a SECA balance scale to the nearest 0.1 kg. Body mass index (BMI) was calculated as weight (kg) divided by the square of the height (m). Obesity was defined as a BMI value higher than the 97th percentile value for age and gender, using the definition and reference values proposed by the International Obesity Task Force in Childhood (19,20). The degree of obesity was quantified using Cole’s least mean square method, which normalizes BMI skewed distribution and expresses BMI in standard deviation scores (SDS). This measure gives age- and sex-specific estimates for median of distribution, coefficient of variation and degree of skewness by a maximum-likelihood fitting technique (20). Control subjects were chosen from among those having a BMI below 85th percentile for age and gender. None of the participants was receiving any medical treatment or had a history or evidence of metabolic, cardiovascular, respiratory or hepatic disease. Pubertal development was assessed according to Tanner criteria (21) and the children were evaluated as prepubertal and pubertal.

Fasting plasma glucose, serum triglyceride (TG), total cholesterol (TC) and high-density lipoprotein-cholesterol (HDL-C) concentrations were measured enzymatically using an autoanalyzer (Olympus 2700, Olympus Medical Systems Corp. Tokyo, Japan). The low-density lipoprotein-cholesterol (LDL-C) level was calculated using the Friedewald equation. Plasma insulin was measured by the electrochemiluminescence immunoassay method using an automated immunoassay analyzer (E170, Roche, Hitachi, Osaka, Japan). The children and their parents had been carefully instructed that a fasting period of at least 12-14 hours should be provided. Cut-off points above the 95th percentile of healthy children were used to determine dyslipidemia and impaired fasting glucose according to international recommendations (22,23).

In the obese group, insulin sensitivity was determined by using the homeostasis model assessment for insulin resistance (HOMA-IR). HOMA-IR was calculated using the equation: HOMA-IR=fasting insulin (μU/mL) x fasting glucose (mg/dL)/405. Obese children were subdivided according to their HOMA-IR values as insulin-resistant and non-insulin-resistant. Insulin resistance was defined as HOMA-IR values above 2.67 and 2.22 for prepubertal boys and girls, respectively, and above 5.22 and 3.82 for pubertal boys and girls in the same order (24).

**Heart Rate Variability  **

Twenty-four-hour ambulatory electrocardiographic recordings were obtained from each subject with Rozinn RZ 152 digital Holter recorder (Rozinn Electronics, Inc., Glendale, NY, USA), with a sampling frequency of 1024 Hz. All cases were strictly advised to maintain the normal course of their daily life. Their compliance was confirmed again while removing the device from the cases. The HRV was determined by the software of the same device.

The time-domain HRV measures employed in our study were as follows:  standard deviation values of all normal sinus R-R intervals over 24 hours (SDNN); standard deviation values of all averaged normal sinus R-R intervals for each 5-minute segment in the 24-hour recordings (SDANN); root mean squares of successive differences between normal sinus R-R intervals (RMSSD); ratios of number of all R-R intervals to height of the histogram created by charting all R-R intervals (HRV triangular index); numbers of R-R intervals exceeding 50 ms (SNN50 count);  percentage of difference between adjacent normal R-R intervals that are greater than 50 ms computed over the entire 24-hour ECG recording (PNN50). Among them, RMSSD, SNN50, and PNN50 primarily reflect parasympathetically mediated changes in heart rate (25). Other time-domain variables reflect a mixture of parasympathetic, sympathetic, and other physiologic influences. 

We used power spectral analysis of heart rate with parameters of low frequency (LF: 0.04-0.15 Hz) which are related to baroreceptor control and are dually mediated by vagal and sympathetic systems, as well as those of high frequency (HF: 0.15 – 0.5 Hz) which reflect respiratory sinus arrhythmia and thus, cardiac vagal activity (26,27). Also, the most indicative parameter of LF/HF was used for assessing the autonomic balance. 

## STATISTICAL ANALYSIS

We used SPSS for Windows, Version 15 software (SPSS Inc., Chicago, IL., USA) in the analyses. The Shapiro-Wilk test was used to test for normality of variables. The decision to perform parametric testing was based on the result of this test for continuous variables. Comparisons between the groups were carried out with two-tailed Student’s t-test for normally distributed continuous variables, Mann-Whitney U test for continuous variables without normal distribution, and chi-square test for dichotomous variables. P values below 0.05 were considered as significant.

## RESULTS

In the study group, 8 (25%) children had hypertriglyceridemia, 4 (13%) hypercholesterolemia, 2 (6%) increased LDL-C levels, 6 (19%) decreased serum HDL-C levels, and 21 (66%) showed impaired fasting glucose. None of the lean subjects had impaired fasting glucose.

There was no significant difference with regard to age and gender between the study group and the control group (p=0.857 and p=0.578, respectively). Demographic,anthropometric, and biochemical characteristics of the participants are given in Table 1. As seen in the Table, the obese group had significantly higher insulin, TG, VLDL-C and lower HDL-C levels than the control group.

HRV indices for both time-domain and frequency-domain and their statistical comparison between the groups are illustrated in Table 2. The study and control groups were found to be statistically different in all parameters, except SDNN. While SDANN, RMSSD, NN50 index, NN50 count, PNN50, HRV-triangular index, LF and LF/HF were significantly lower in the study group than in the control group, the HF was found to be significantly higher in the study group than in the control group. 

When we categorized the study group according to their HOMA-IR values, 21 children (14 male, 7 female) were found to have insulin resistance, while 11 (6 male, 5 female) did not. The comparisons of HRV values between insulin-resistant and non-resistant subgroups are shown in Table 3. 

**Table 1:**
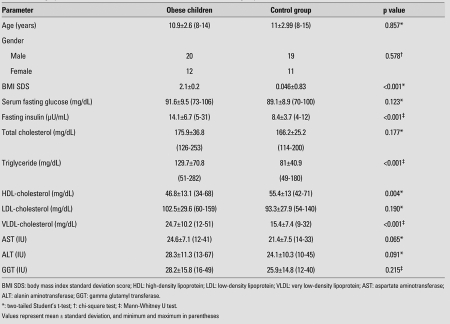
Demographic, clinical and biochemical values in the obese and control groups

**Table 2:**
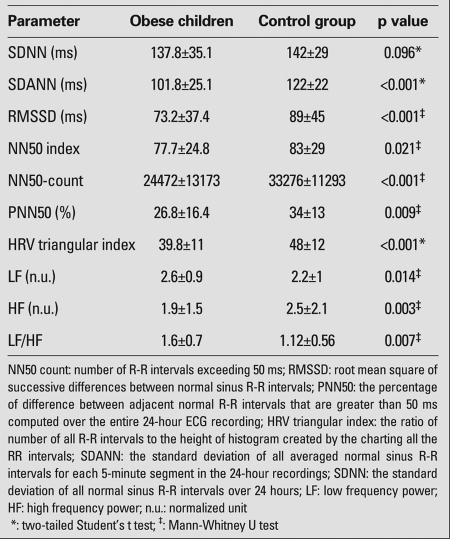
Time-domain and frequency-domain parameters of the obese and control children

**Table 3:**
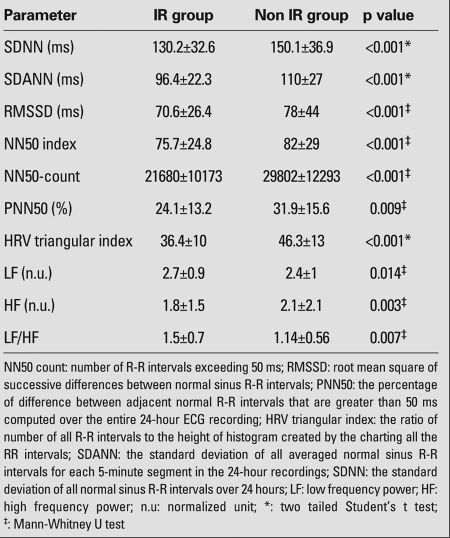
Time-domain and frequency-domain parameters and their  comparison between insulin resistant (IR) and non-insulin resistant  (Non IR)  groups

## DISCUSSION

The results of our study show that both time-domain and frequency-domain parameters of HRV were decreased in obese children - a finding, reflecting parasympathetic withdrawal and sympathetic predominance. Also, the subgroup analysis of the study group demonstrated a marked parasympathetic withdrawal and resulting sympathetic predominance in the insulin-resistant group as compared to the group with no resistance to insulin. 

Using power spectral analysis, Piccirillo et al (8) reported that obesity was associated with decreased sympathetic responsiveness. Obese subjects showed a higher presynaptic activation level as indicated by plasma norepinephrine levels. At the same time, postsynaptic sympathetic responsiveness was diminished in these subjects. The decreased sympathetic reactivity to stress was thought to be a contributing factor to the higher mortality rates. A low sympathetic activity was also reported by Peterson et al (28).

Rossi et al (6) reported a lower parasympathetic function in obese subjects, but found no differences in sympathetic functions between normal and obese individuals. The former finding was suggestive of a causal role of parasympathetic tone in sudden death. A decreased parasympathetic activity has also been reported by Arone et al (29). However, in contrast to other studies, these authors found also an increase in the sympathetic control. Zahorska-Markiewicz et al (30) observed overreactivity of the sympathetic nervous system, as opposed to a depression in parasympathetic activity analogous to that reported by Piccirillo et al (8). A study by Arone et al (29) showed an increase in parasympathetic activity with weight loss and an inverse behavior of sympathetic drive in moderately overweight subjects, but these results were based on short-term differences in a population with rather fast weight changes. In a study on weight gain, Hirsch et al (31) described an inverse relationship between amount of weight gain and lower parasympathetic drive. In obese women, Gao et al (32) reported higher sympathetic and parasympathetic activity, especially when there was a combination of upper body obesity and visceral obesity. The limited studies conducted in obese children indicate parasympathetic withdrawal and sympathetic predominance with metabolic changes such as impaired blood lipid profile (33,34,35). 

The results of our study were in line with the above-mentioned reports. Our findings revealed reduced parasympathetic activity resulting in sympathetic predominance. However, in evaluating the autonomic nervous system, we used only time-domain and frequency-domain parameters of HRV; therefore, we cannot report any conclusions pertaining to the mechanism of this cardiac autonomic imbalance. This point should be evaluated by future large-scale prospective studies using arterial blood pressure variability and skin tests. However, an important finding of our study is that insulin-resistant obese children have marked sympathetic predominance compared to their non-insulin-resistant counterparts. Whether insulin resistance causes the autonomic imbalance or not is not clear. Theories about this point generally focus on the question of whether autonomic imbalance is the key factor leading to insulin resistance, hypertension and metabolic syndrome or not (36). 

In conclusion, we found sympathetic dominance in the obese children, which was more significant in the insulin-resistant subgroup. Large-scale prospective studies are needed to delineate the exact mechanisms that take part in the interactions between obesity, insulin resistance and autonomic functions.
